# Factors affecting the implementation of a complex health intervention to improve insulin management in primary care: A SWOT analysis

**DOI:** 10.4102/phcfm.v14i1.3467

**Published:** 2022-07-04

**Authors:** Patrick Ngassa Piotie, Celia Filmalter, Maryangela G. Mohlala, Ntokozo Zulu, Amanda Segale, Charles Koenaite, Jane W. Muchiri, Elizabeth M. Webb, Paul Rheeder

**Affiliations:** 1 School of Health Systems and Public Health, Faculty of Health Sciences, University of Pretoria, Pretoria, South Africa; 2 University of Pretoria Diabetes Research Centre Faculty of Health Sciences, University of Pretoria, Pretoria, South Africa; 3 Department of Nursing Science, Faculty of Health Sciences, University of Pretoria, Pretoria, South Africa; 4 Department of Human Nutrition, Faculty of Health Sciences, University of Pretoria, Pretoria, South Africa; 5 Department of Internal Medicine, Faculty of Health Sciences, University of Pretoria, Pretoria, South Africa

**Keywords:** SWOT analysis, diabetes, type 2 diabetes, insulin management, health intervention, primary care, telehealth, community health workers

## Abstract

**Background:**

In South Africa, initiating and managing insulin in primary care for people living with type 2 diabetes (PLWD) is a major challenge. To address these challenges, a multidisciplinary team from the University of Pretoria (South Africa) developed the Tshwane Insulin project (TIP) intervention.

**Aim:**

To determine internal and external factors, either facilitators or barriers, that could influence the implementation of the TIP intervention and propose strategies to ensure sustainability.

**Setting:**

Tshwane District, Gauteng province, South Africa.

**Methods:**

We used the SWOT framework to qualitatively analyse the strengths, weaknesses, opportunities, and threats influencing the implementation of the TIP intervention. Four field researchers and three managers from the TIP team participated in an online group discussion. We also conducted semi-structured interviews with healthcare providers (HCPs) (seven nurses, five doctors) and patients with type 2 diabetes (*n* = 13).

**Results:**

Regardless of the identified weaknesses, the TIP intervention was accepted by PLWD and HCPs. Participants identified strengths including app-enabled insulin initiation and titration, pro-active patient follow-up, patient empowerment and provision of glucose monitoring devices. Participants viewed insulin resistance and the attitudes of HCPs as potential threats. Participants suggested that weaknesses and threats could be mitigated by translating education material into local languages and using the lived experiences of insulin-treated patients to address insulin resistance. The procurement of glucose monitoring devices by national authorities would promote the sustainability of the intervention.

**Conclusion:**

Our findings may help decision-makers and health researchers to improve insulin management for PLWD in resource-constrained settings by using telehealth interventions.

## Introduction

The treatment of type 2 diabetes mellitus (T2DM) has moved from specialist centres to primary care settings.^1,2^ To sustain this move, healthcare providers in primary care settings must effectively provide aspects of diabetes care traditionally offered by specialists. For instance, primary care providers are becoming increasingly responsible for the appropriate management of insulin in T2DM.^2^ Insulin management in primary care faces several challenges including initiation, optimisation and intensification of insulin therapy.^1,3,4,5^

In South Africa, initiating and managing insulin for people living with diabetes (PLWD), especially type 2, are major challenges considering the limited resources of the healthcare system, especially in primary care. These system challenges are exacerbated by patient beliefs and resistance towards insulin and the attitudes of healthcare providers who prefer to delay insulin therapy in spite of patients not achieving glycaemic goals.^6,7,8^ Consequently, people with T2DM often remain on maximum oral glucose-lowering therapy for unnecessarily long periods and people who have switched to insulin from oral drugs rarely optimise or intensify their insulin therapy.^9^ Proper insulin management also requires consumables such as syringes, blood glucosemonitors and test strips.^10^ In South Africa, blood glucose monitors and test strips for self-monitoring of blood glucose are limited and seldom used by PLWD in public healthcare settings.^11^

To address these challenges related to insulin management in primary care, a multi-disciplinary team from the University of Pretoria in South Africa developed a nurse-driven and home-based telehealth intervention named the Tshwane Insulin project (TIP).^12^ The TIP intervention was developed using the Integrated Chronic Disease Management (ICDM) framework^13^ and comprises a facility-level intervention, where professional nurses evaluate PLWD and initiate insulin, an individual-level intervention where community healthcare workers (CHWs) monitor patients at their homes and provide educational information, whilst using telehealth to enable physician-directed insulin titration if needed, and a community-level intervention aimed at empowering CHWs to support PLWD and raise awareness of diabetes.^12^

The TIP intervention was developed in four sequential phases following the guidance of the United Kingdom (UK) Medical Research Council (MRC) for designing and evaluating complex interventions.^14^ The four phases were (I) planning, (II) design, (III) implementation and (IV) evaluation.^12^ During phase IV, the TIP intervention was piloted at 10 primary care facilities in the Tshwane District. Patients with T2DM and primary health care providers including doctors, nurses and CHWs were involved in the trial. The findings from the pilot study are published elsewhere.^15^ After the pilot, the researchers conducted a Strengths, Weakness, Opportunities and Threat (SWOT) analysis to identify the strengths (S) and weaknesses (W) of the intervention, as well as opportunities (O) to be exploited and threats (T) that must be avoided to ensure the success of the TIP during large-scale implementation. A SWOT analysis is an effective planning tool to identify the intrinsic (S and W) and extrinsic (O and T) factors that need to be considered to achieve success.^16^ Strengths, Weakness, Opportunities and Threat analyses have been used to evaluate health interventions and to enhance performance and service quality.^17,18^ It can also be used to evaluate interventions to gain an early idea of possible future consequences.^17^

Currently, there is limited research on the successful implementation and dissemination of telehealth interventions to address insulin initiation and titration in primary care in resource-constrained settings. Here, we report on the internal and external factors, either facilitators or barriers, identified using a SWOT analysis that could affect the implementation and scale-up of the TIP intervention. Based on participants’ feedback, we propose strategies to facilitate the implementation and dissemination of the intervention and ensure its sustainability.

## Methods

### Study design

We conducted a SWOT analysis using a qualitative design. The SWOT methodology is structured and allows for a qualitative descriptive and cross-sectional analysis.^19^ This study had two components: (1) an online focus group discussion (FGD) with the members of the TIP team involved in implementing the intervention and (2) semi-structured interviews with PLWD and healthcare providers who participated in the pilot study (results reported elsewhere).

### Setting

The study was conducted in the Tshwane District, which is situated in the northern part of the Gauteng province in South Africa. The population is urban and consists of mixed socioeconomic groups living in traditional and informal dwellings. People who attend the primary care clinics are often from middle to low socioeconomic groups and cannot afford medical insurance. The primary care clinics where the study took place were selected conveniently during the pilot of the TIP intervention (reported elsewhere).

The prevalence of diabetes mellitus in the Tshwane District is unknown; however, suboptimal control for blood glucose and blood pressure as well as high rates of complications have been reported in people with diabetes living in this district.^20^ Most PLWD must visit their local primary care clinic at least four times a year to be seen by professional nurses and occasionally medical officers.^21^ In practice, most patients with T2DM attend clinics monthly to collect their medication and have their blood glucose, blood pressure and weight checked.^11^ South Africa adopted a stepwise approach to the management of T2DM. Patients with T2DM are first managed with glucose-lowering tablets and lifestyle modifications. Insulin therapy is recommended for people with T2DM who are not controlled (HbA1c > 7%) despite lifestyle modifications and adherence to a combination of oral drugs.^22^

### Study population

For this study, we used criterion sampling as a purposive sampling strategy.^23^ Criterion sampling, specifically criterion-i sampling, was used because the study participants: (1) had to have been involved in the implementation of the TIP intervention, (2) had to possess knowledge and experience with the implementation process and (3) had to be able to provide information that is both detailed and generalisable.^23^

After the pilot of the TIP intervention, participants comprising primary care nurses and doctors who were involved in the pilot study and PLWD who were initiated on insulin during the pilot and completed a 14-week follow-up were invited for semi-structured interviews. In addition to the pilot study participants, members of the TIP team were invited to take part in an online FGD. The TIP members were included in the study because they possessed valuable information that had to be included in the evaluation of the strengths, weaknesses, opportunities and threats of the TIP intervention and the implementation process.

We achieved data saturation (1) by including all available participants, (2) by interviewing participants who had different perspectives and (3) by exploring the insights and experiences of doctors, nurses, patients as well as the TIP staff with the TIP intervention and the implementation process.^23^

### Data collection

#### Individual semi-structured interviews

Healthcare providers and patients with T2DM who were involved in the TIP intervention pilot were interviewed about the benefits and disadvantages of the intervention. The questionnaire was adapted from tools developed by Weiner et al.^24^ Six Likert-scale statements assessed satisfaction and acceptability of the intervention, whilst two open-ended questions focused on the challenges encountered as well as suggestions for improvement. The questionnaires were administered in English by trained fieldworkers who met with healthcare providers at their workplace and with PLWD at their home or their local clinic. The interviews took place from April to June 2021 and were audio recorded.

#### Focus group discussion

We held an online FGD with members of the TIP team in July 2021. The online FGD was facilitated by CF. All participants provided written informed consent. The FGD took 4 h and followed the SWOT analysis framework of first identifying strengths followed by weaknesses, opportunities and lastly threats. The FGD was done on Microsoft Teams and was recorded.

### Data processing and analysis

Data from the FGD and the semi-structured interviews were transcribed verbatim by PNP. Then, PNP and CF individually analysed the data manually using the SWOT framework.^17^ CF is an academic with vast experience in qualitative research, whilst PNP is a junior researcher who has been exposed to qualitative research. Themes were identified from the transcripts and were analysed and interpreted within a SWOT framework. PNP and CF then had a meeting to compare, regroup and plot the themes onto the SWOT matrix. PNP anonymised relevant excerpts and added them to the SWOT matrix. The plotted themes and excerpts were sent to the FGD participants for member checking, and consensus was reached on the themes. Lastly, JWM who is an experienced qualitative researcher reviewed the work. The researchers had no relationship with the participants who were interviewed.

### Ethical considerations

The Research Ethics Committee of the Faculty of Health Sciences of the University of Pretoria (Ethics Reference No.: 156/2019) and the Tshwane Research Council (No.: GP_201810_049) approved the study. The study adhered to the Declaration of Helsinki. Written informed consent was obtained from all participants.

## Results

A total of 31 people participated in the study. Twenty-five participants comprising seven primary care nurses and five medical officers who were involved in the pilot, and 13 PLWD accepted the invitation and were interviewed. In addition, six members of the TIP team took part in the FGD: three were part of the management team, whilst the remaining three were field researchers.

The internal aspects (strengths and weaknesses) that characterised the TIP intervention are summarised in Table 1.

**TABLE 1:** Strengths and weaknesses (internal aspects) of the Tshwane Insulin Project intervention identified by members of the Tshwane Insulin Project team, healthcare providers and patients.

Strengths	Weaknesses
Promote better diabetes care for PLWD, reduce referrals for insulin initiation and improve patient outcomes.	Education materials written in English. Complicated follow-up procedure for working patients.
Pro-active patient follow-up and support.	
Empowerment and education of PLWD and their families.	
Provision of monitoring equipment, consumables and education materials.	
Innovative telehealth intervention embedded in the ICDM framework.	Reliance on smartphones and on the availability of mobile data and network.
App-enabled initiation and titration of insulin.	
Involvement of CHWs with home visits.	Perceived increased workload for clinic staff.
Simplified protocols and processes aligned to NDOH diabetes management guidelines.	
Empowerment and training of healthcare providers, sharing best practices.	
Strong engagement with stakeholders and active consultation.	
Fostering collaboration amongst healthcare providers.	
Knowledgeable and competent field researchers.	

CHW, community healthcare workers; ICDM, Integrated Chronic Disease Management; PLWD, People living with diabetes; NDOH, National Department of Health.

### Strengths of the Tshwane Insulin Project intervention

#### Provision of monitoring equipment, consumables and educational material

After initiating insulin, enrolled patients received a pack containing various items including a blood glucose monitor, test strips, lancets for finger pricking, a diabetes education booklet, a diary for self-monitoring of blood glucose and a sharp container for disposing insulin needles and lancets. Outside of the TIP, patients who are initiated on insulin in South African primary care facilities do not receive glucose monitors and strips. A healthcare professional noted:

‘Mostly I like the fact that patients are given strips and meters for monitoring, because the department [*of health*] don’t provide them. Also, the involvement of CHWs is good.’ (Participant 4, female, nurse)

#### Pro-active patient follow-up and support

After initiating insulin, patient follow-up was not limited to monthly clinic visits. In the TIP, patients were visited weekly for 14 weeks by CHWs and TIP field researchers. During home visits, CHWs checked on injection sites and injection technique, monitored home glucose measurements, checked on compliance with the clinic visit schedule and checked for hypo and hyperglycaemia. The CHWs delivered individualised patient education based on patient needs using the diabetes education booklet. Enrolled participants appreciated the additional support:

‘I liked the fact that, they were checking me every week. They gave me machine and strips and they also provided voucher for food parcel.’ (Participant 8, PLWD, female)

#### Empowerment and education of people living with diabetes and their families

Patients who participated in the TIP intervention received adequate education before initiating insulin followed by education sessions during home visits. The sessions focused on improving patient knowledge and understanding of insulin therapy and helping patients to adopt self-care behaviours such as healthy eating, being physically active and monitoring blood glucose. Additionally, home visits encouraged family involvement. Family members were more supportive after attending education sessions. A participant commented:

‘TIP helped me. I can now control my glucose levels. Before TIP, I had no information now I can control my diet through the knowledge I gained.’ (Participant 12, PLWD, female)

#### App-enabled initiation and titration of insulin at clinics and homes

The TIP enables primary care nurses to use a mobile app called Vula to connect with a doctor to initiate a patient on insulin. In this setting, PLWD often have limited numeracy skills, health literacy and limited understanding of diabetes, which means that self-titration was not a realistic option. Weekly physician-directed titration enabled by telemedicine and assisted by a CHW allowed participants to reach their optimal insulin dose quicker. At each home visit, CHWs used a mobile app to share the home glucose values of the participants with a doctor who indicated via the app whether the current insulin dose should be increased, decreased or remain unchanged. The ability to titrate insulin remotely was a positive experience for the nurses as demonstrated by the following quote:

‘I like the way the patients are followed up at home. And patients [*insulin*] dosage can be changed whenever is necessary.’ (Participant 5, female, nurse)

#### Empowerment of healthcare providers and improved diabetes care for people living with diabetes

Healthcare providers received training in integrated diabetes management and care, which was supplemented by on-the-job training and ongoing support by TIP field researchers. Healthcare professionals became confident in their ability to manage PLWD and to initiate insulin therapy. More patients were initiated on insulin at participating clinics, and the management of PLWD improved with better monitoring (more HbA1c tests done) and optimisation of therapy for patients on oral glucose-lowering drugs. A primary care doctor remarked:

‘The programme is beneficial for the overall management of Type 2 Diabetes and not only the initiation of insulin.’ (Participant 1, male, doctor)

#### Competent field researchers providing technical assistance to healthcare providers and people living with diabetes

TIP field researchers who were qualified healthcare workers were employed full time and received training in diabetes management and care. The field researchers were assigned to primary care clinics to support PLWD. They would often act as diabetes educators, educating patients on diabetes self-management. The field researchers also assisted healthcare providers in identifying qualifying patients and providing pre-initiation counselling. The field researchers developed good interpersonal relationships and rapport with the healthcare workers, which eased the implementation of the intervention.

#### Integrated digital health intervention

The TIP intervention was designed to fit into the ICDM model, which facilitated its integration within the primary South African healthcare system. The intervention showed that inter-professional team-based diabetes care was possible with the help of digital tools such as mobile applications.

### Weaknesses of the Tshwane Insulin Project intervention

#### Education materials written in English constituted a language barrier

The diabetes education booklets distributed to healthcare workers and PLWD were written in English. The TIP education materials were developed in English because many local languages are used in communities where the TIP was implemented, and English seemed to be a practical option. Our SWOT analysis revealed that choosing English constituted a language barrier as many participants were not fluent in English as evidenced by the following statement:

‘To have the booklet in multiple languages that can be understood by many people.’ (Participant 6, PLWD, male)

#### Reliance on smartphones and on the availability of mobile data and network

Although health workers received smartphones and mobile data to use the mobile app, access to mobile networks was sometimes a challenge. Unfortunately, some areas had poor network coverage. Poor network coverage resulted in delays and in some instances insulin titration could not be done from the patient’s home. A health professional noted that:

‘It is a great programme but a problem when there is no network coverage for Vula [*mobile app*] the initiation visit takes long.’ (Participant 3, female, nurse)

#### Perceived increased workload for healthcare providers

The increased workload was identified as a weakness of the TIP as activities were perceived to be time consuming by some primary care nurses, whose main tasks are to identify patients with suboptimal control, refer patients to a doctor for initiation and counsel patients:

‘Time consuming, because I had [*to*] see other patients in waiting.’ (Participant 3, female, nurse)

#### Follow-up of employed patients

In addition, patients who were employed full time did not benefit from home visits. The field researchers of the TIP attempted telephonic follow-ups with calls or WhatsApp; however, the procedure needs to be formalised and strengthened.

The external factors, including the opportunities and threats as identified during the SWOT analysis, are summarised in Table 2.

**TABLE 2:** Opportunities and threats (external factors) of the Tshwane Insulin Project intervention identified by members of the Tshwane Insulin Project team, healthcare providers and patients.

Opportunities	Threats
Presence of a large pool of insulin-requiring PLWD at primary care facilities.	Insulin resistance from PLWD and healthcare providers.
Utilise lived experiences to advocate for insulin therapy amongst PLWD and promote better diabetes care.	Family and community misconceptions and stigmatisation.
Family on-boarding programme before insulin initiation.	Low patient health literacy.
Procurement of monitoring equipment and consumables such as glucose meters and test strips for PLWD in primary care.	Attitudes of healthcare providers.
Strengthen follow-up strategy for employed PLWD.	Poor compliance with diabetes management guidelines.
Translation of education materials.	Doctors not responding on time via the mobile app.
Involvement and training of allied healthcare workers.	Incomplete or unreliable patient records (incorrect contact details).
Involvement of CHWs in diabetes care.	Health workforce challenges: staff rotation or turnover, overworked and overburdened health workforce.
Larger number of doctors registering on the digital health platform.	Poor filing and data systems at primary care facilities.
Employment of Diabetes Educator or Dedicated Diabetes Nurses.	Shortages of insulin, monitoring equipment and consumables.
Data-free mobile app for initiation and titration of insulin.	Competing priorities at the primary care facilities.
Wi-Fi roll-out at primary care facilities.	Repeat prescriptions with limited monitoring and follow-up.
Scaling-up, dissemination of the intervention.	Limited capacity and human resources for patient recruitment, counselling and follow-up.

PLWD, people living with diabetes; CHWs, community health workers.

### Opportunities of the Tshwane Insulin Project intervention

#### Task sharing and involvement of allied healthcare workers

The various tasks of the TIP intervention can be shared amongst healthcare workers to ease the load on primary care nurses. For example, health promoters could be trained and be responsible for counselling patients who need to be initiated on insulin. A nurse suggested the following:

‘I would suggest that TIP train people like health promoters so that they can do the counselling of diabetes patients.’ (Participant 7, male, nurse)

#### Involvement of community healthcare workers in diabetes care

The TIP intervention is an opportunity to train CHWs to care for PLWD. The CHWs are able to reach out to patients in their homes and deliver basic educational information that reinforces the health education received from nurses or doctors during clinic visits. During the pilot, PLWD enjoyed the home visits by CHWs. However, the visits were stopped after the end of the 14-week follow-up period. A participant remarked:

‘Home visits are good, but they stopped after discharge [*at the end of the pilot study*].’ (Participant 9, PLWD, female)

#### Employment of dedicated diabetes nurses or diabetes educators

The TIP highlights the need for dedicated diabetes nurses or diabetes educators to support healthcare providers in managing PLWD. The TIP field researchers often played the role of dedicated diabetes nurses or educators that was not sustainable. In the South African public healthcare system, staff rotation and turnover threaten the quality of care given to patients. Dedicated diabetes nurses will promote continuity of care, and they will be able to educate and counsel patients and prepare patients for insulin therapy.

#### Scaling-up of the intervention

The TIP intervention could be replicated in other parts of South Africa and in other resource-constrained settings. The TIP intervention fits into the ICDM model adopted by the National Department of Health to achieve optimal outcomes for patients with chronic communicable and non-communicable diseases. There is a large pool of insulin-requiring T2DM patients who would benefit from the TIP intervention, and PLWD who have already been initiated on insulin could provide peer support to those patients. The dissemination of the TIP intervention was supported by health professionals involved in the pilot:

‘I wish it can [*be*] implemented everywhere within South Africa, all other provinces. Especially the rural areas.’ (Participant 10, female, doctor)

### Threats of the Tshwane Insulin Project intervention

#### Insulin resistance from people living with diabetes

During the pilot study, a number of patients refused to start insulin despite being encouraged to do so by healthcare providers. Insulin resistance was common amongst PLWD, and negative attitudes towards insulin may have been fuelled by family or community misconceptions about insulin. This was corroborated by a health professional:

‘Most of the patients have fear of starting insulin.’ (Participant 2, female, nurse)

#### Attitudes of healthcare providers and dependence on the field researchers

Some healthcare providers failed to take ownership of the intervention because they believed that it was not their duty to complete the prescribed tasks, even though initiating patients on insulin is their responsibility according to the guidelines for the management of diabetes in primary care. At times, healthcare providers relied heavily on TIP field researchers to complete certain tasks. Such attitudes threatened the sustainability of the TIP intervention. A nurse commented:

‘Having TIP people like *Thabo* [*TIP field researcher – pseudonym*] every day in the clinic helps, because if they are not around, we forget to do certain things.’ (Participant 5, female, nurse)

#### Doctors not responding on time via the app

Additionally, the successful use of telehealth requires doctors to be available on the mobile app when the nurses need to reach them. Not being available is a threat to the TIP. Nurses could not always reach doctors timeously, resulting in frustration whilst waiting for a doctor to respond, which also meant a longer consultation time for the patient. A nurse explained:

‘When the clinic doctor was unavailable it was difficult to get hold of family doctor [*physician*] at the referring hospital. It ended up taking more time than was necessary and patients were discouraged as they had to spend more time at the clinic or they left the clinic without their insulin treatment as promised.’ (Participant 11, female, nurse)

#### Overworked and overburdened health workforce

In South Africa, primary healthcare workers routinely deal with high patient volumes and a quadruple burden of disease. These factors have been shown to result in short consultation times, lack of dedicated staff for patient education and the pressure to see patients quickly and have also been identified as barriers to insulin initiation. In this context, providers may hesitate to implement the TIP intervention as demonstrated by the following quote:

‘I would suggest that because the programme has its own clinicians they can be able to initiate and manage patients to reduce lot of work for clinic nurses.’ (Participant 13, female, nurse)

#### Health system factors

Participants identified a number of health system factors as potential threats to the implementation of the TIP intervention. Those included poor filing and data systems, incomplete or unreliable patient records which make patient identification difficult and cumbersome; limited resources including a shortage of insulin or needles and competing priorities at primary care facilities.

#### Strategies derived from the strengths, weaknesses, opportunities and threats analysis

After gathering data according to the SWOT categories recorded in Tables 1 and 2, the main themes were analysed to formulate the strategies contained in Table 3. For example, SO (strengths-opportunities) strategies rely on internal strengths to take advantage of opportunities, whilst WO (weaknesses-opportunities) strategies introduce new opportunities to reduce weaknesses. Through ST (strengths-threats) strategies, threats are avoided by using internal strengths, and WT (weaknesses-threats) strategies minimise internal weaknesses and avoid threats.

**TABLE 3a:** Strengths, weakness, opportunities and threat analysis strategies to improve the implementation of the Tshwane Insulin Project intervention.

SO strategies	WO strategies
1. Replicate the TIP intervention to respond to the existing large pool of insulin-requiring PLWD at primary care facilities and avoid unnecessary referrals for insulin initiation.	1. Translate the education materials to improve accessibility by PLWD and improve health literacy.
2. Promote the use of simplified protocols and processes to improve healthcare providers’ confidence to initiate insulin safely.	2. Roll out good quality Wi-Fi at all primary care facilities to enable digital health interventions including the TIP intervention and to resolve issues related to the unavailability of mobile data and network.
3. Adopt a multidisciplinary care team approach to facilitate insulin initiation and titration and ensure that primary healthcare providers share the workload.	3. Hire dedicated diabetes nurses or diabetes educators to alleviate providers’ workload and ensure the delivery of good diabetes care including initiation of insulin.
4. Develop strategies to support insulin-requiring PLWD such as a family on-boarding programme, dramatic plays or informative sessions with insulin-using PLWD.	4. Involve allied healthcare workers to reduce the pressure on providers by sharing tasks such as patient education and counselling.
-	5. Involve WBOT/CHWs in the follow-up of employed patients, explore home visits on weekends.

TIP, Tshwane Insulin Project; SO, strengths-opportunities; WO, weaknesses-opportunities; PLWD, people living with diabetes; WBOT/CHWs, ward-based outreach team/community health workers.

**TABLE 3b:** Strengths, weakness, opportunities and threat analysis strategies to improve the implementation of the Tshwane Insulin Project intervention.

ST strategies	WT strategies
1. Educate PLWD using patients’ lived experiences to combat insulin resistance and advocate for insulin therapy amongst PLWD and in communities.	1. Educate patients and their families by providing culturally-sensitive education materials, to address community misconceptions and stigma attached to diabetes and insulin.
2. Strengthen the engagement with stakeholders including healthcare providers to secure their buy-in and improve their adoption of new technologies.	2. Resolve health workforce challenges as well as issues with mobile data and network to ensure buy-in and participation of healthcare providers.
3. Procure monitoring equipment and consumables for PLWD to promote insulin initiation in primary care and to avoid discontinuation of insulin therapy.	-
4. Strengthen the training of healthcare providers to ensure compliance with guidelines and avoid detrimental practices such as the abuse of repeat prescriptions with limited monitoring of PLWD.	-

ST, strengths-threats; WT, weaknesses-threats; PLWD, people living with diabetes.

Whilst the TIP team may be able to translate educational materials into local languages or educate patients by sharing lived experiences of PLWD initiated on insulin, other strategies will require the commitment and support of the health authorities. For example, the National Department of Health will have to procure monitoring equipment (glucometers and test strips) for all insulin-treated PLWD in primary care. The roll out of good quality Wi-Fi at primary care facilities and the provision of smartphones to health workers is also part of the infrastructure that needs to be provided by health authorities.

## Discussion

The future of diabetes care in resource-constrained settings lies in the decentralisation of care, from experts who work in hospitals to CHWs and other non-clinical providers who work in the primary care system to deliver home-based screening and care enabled by simple and effective information technology solutions.^25^ The TIP intervention demonstrates that transforming the delivery of care is possible in sub-Saharan Africa. However, transferring new knowledge from public health research into practice is a very complex process.^26^ Hence, we adopted a phased approach to evaluate the TIP intervention and understand the implementation challenges using the UK MRC framework.^27^ A SWOT analysis combined with traditional qualitative research provided insights into the views and perspectives of different stakeholders, including healthcare professionals and PLWD who participated in the TIP intervention.^28^ We identified positive and negative factors, and we formulated a number of strategies to improve the implementation and dissemination of the intervention.

Our SWOT analysis suggests that, regardless of the identified weaknesses and potential threats, the TIP intervention was accepted by PLWD and healthcare providers involved in the pilot study. According to the participants, the TIP intervention benefitted and improved insulin management for patients with T2DM. People living with diabetes who participated in the TIP were also more knowledgeable about their condition. Insulin-treated PLWD who feel empowered by healthcare providers might have an increased sense of control of their condition and, subsequently, might become more actively involved in disease management, resulting in better glycaemic control.^29^

At the time when insulin is presented as an option, PLWD often experiences insulin distress, defined as an emotional response to the suggested use of insulin characterised by severe apprehension, discomfort, dejection or denial because of a perceived inability to cope with the requirements of insulin therapy.^5^ In the TIP intervention, insulin distress was overcome by patient education, through pro-active follow-up at primary healthcare clinics and during weekly home visits. Effective communication and pro-active contact contribute to successful insulin initiations.^30,31^

McGloin et al.^32^ acknowledged that providing adequate support and monitoring when PLWD transition from oral drugs to insulin can present challenges for healthcare providers, particularly between healthcare visits. The TIP intervention introduced home visits for T2DM patients as they transition to insulin to maintain patient-provider contact. Newly initiated PLWD appreciated the home visits, where telehealth created additional opportunities for insulin titration. Telemedicine or telehealth makes it easier to care for people living with chronic diseases such as diabetes and enables healthcare providers to deliver better care.^33^ Polonsky et al.^31^ recommend titration strategies including the use of technology when the patient is unable to calculate the appropriate dose as is often the case in our setting. Beyond enhanced support including assisting with insulin titration, telemedicine strategies help patients with T2DM to adhere to insulin regimens,^30^ and are associated with improved glycaemic control.^34,35^

In the pilot study, participants felt that the supply of equipment to monitor blood glucose was particularly helpful. Access to blood glucose monitors and test strips enhance patient education and empowerment,^25^ and PLWD who cannot afford test strips and are inexperienced in insulin dosage are more likely to discontinue insulin therapy.^36^ The procurement of monitoring equipment and consumables for PLWD by the National Department of Health should promote insulin initiation in primary care and also lead to patients feeling more empowered and able to participate in their own care.

We identified the negative attitudes of healthcare professionals as a major threat to the TIP intervention. Healthcare professionals are important partners in the implementation of innovative interventions such as the TIP. Therefore, their buy-in and participation are crucial to ensure the success and sustainability of the intervention. Addressing their concerns of a perceived increased workload and lack of time should be a priority. Involving health promoters and hiring dedicated diabetes nurses or diabetes educators could allow healthcare professionals to focus specifically on clinical tasks, reducing their workload. Task sharing has been successfully implemented in South Africa in the human immunodeficiency virus (HIV) programme,^37^ a similar strategy should benefit PLWD and improve insulin management in primary care.

The SWOT analysis resulted in 15 strategies to improve the implementation of the TIP intervention, making it more sustainable. The low-hanging fruits include translating education materials into local languages, developing a strategy to follow-up employed patients and using the lived experiences of insulin-treated patients to address insulin resistance and advocate for insulin therapy amongst PLWD and in communities. Our ultimate aim is to integrate the TIP into primary care across South Africa. To achieve this, the TIP will have to be adopted into national health plans and facilities, garnering a portion of the health budget. An initial investment will result in long-term gains for diabetes management in South Africa.

### Limitations of the study

Subjectivity and unilateralism are amongst the common limitations of a SWOT analysis as the people who determine the factors falling into the four SWOT categories may be too involved to be objective.^38^ The following measures were taken to minimise subjectivity that may influence the results and interpretations: (1) the views and opinions of the recipients of the intervention (healthcare professionals and PLWD) were used to corroborate the findings of the TIP team; (2) the field researchers were involved in the SWOT analysis alongside managers, their valuable experiences enriched the analysis and (3) an independent academic with expertise in qualitative research facilitated the SWOT analysis and contributed to the evaluation of the factors. The inclusion of the TIP members in the SWOT analysis could constitute a limitation because of their position and involvement in the implementation of the TIP intervention; however, their contributions enriched the SWOT analysis and their potential bias was balanced by the involvement of an independent academic.

## Conclusion

The SWOT analysis provided insights on the strengths of the TIP intervention and that strategies could facilitate implementation and dissemination of the TIP intervention. The analysis painted an overall picture of the complexity of implementing innovative health interventions in settings where resources are limited. Lessons learnt from this exercise will assist decision-makers and health researchers in resource-constrained settings who seek to improve insulin management for PLWD in primary care using community-oriented telehealth interventions.
